# Rethinking ratio-based normalization towards model-based approaches in heart weight analysis

**DOI:** 10.1038/s41598-026-43503-x

**Published:** 2026-03-17

**Authors:** Manuela A. Oestereicher, Patricia da Silva-Buttkus, Valérie Gailus-Durner, Susan Marschall, Helmut Fuchs, IMPC Consortium, Jason D. Heaney, Jacqueline K. White, Yann Herault, Masaru Tamura, Kent KC Lloyd, Je Kyung Seong, Lauryl MJ. Nutter, Martin Hrabě de Angelis, Elida Schneltzer, Nadine Spielmann

**Affiliations:** 1 Institute of Experimental Genetics and German Mouse Clinic, Helmholtz Munich, German Research Center for Environmental Health (GmbH), Ingolstädter Landstraße 1, 85764 Neuherberg, Germany; 2 Chair of Experimental Genetics, TUM School of Life Sciences, Technische Universität München, Alte Akademie 8, 85354 Freising, Germany; 3 German Center for Diabetes Research (DZD), Ingolstädter Landstrasse 1, 85764 Neuherberg, Germany; 4 Department of Molecular and Human Genetics, Baylor College of Medicine, One Baylor Plaza, Houston, Texas 77030 United States of America; 5 Center for Biometric Analysis, The Jackson Laboratory, 600 Main Street, Bar Harbor, ME 04609 USA; 6 Université de Strasbourg, CNRS, INSERM, Institute de la Clinique de la Souris, PHENOMIN, 1 rue Laurent Fries, 67404 ILLKIRCH, France; 7 Experimental Animal Division, RIKEN BioResource Research Center, 3-1-1 Koyadai, Tsukuba, Ibaraki 305-0074 Japan; 8 Mouse Biology Program, University of California, 2795 Second Street Suite 400, 50, Davis, California 95618 USA; 9 Korea Mouse Phenotyping Consortium (KMPC) and BK21 Program for Veterinary Science, College of Veterinary Medicine, Research Institute for Veterinary Science, Seoul National University, Seoul, South Korea; 10 The Hospital for Sick Children, Toronto, ON M5G 1X5 Canada

**Keywords:** Cardiology, Computational biology and bioinformatics, Physiology

## Abstract

Heart weight (HW) is a critical parameter in cardiology and mouse research, commonly normalized to body weight (BW) or tibia length (TL) to account for size differences. Ratio-based normalization, however, assumes strict proportionality between variables, an assumption that is rarely tested and may bias group comparisons. We analysed HW, BW, and TL measurements from over 25,000 C57BL/6N wildtype mice generated by the International Mouse Phenotyping Consortium. Sex- and age-stratified analyses were combined with simulation-based modelling to evaluate empirical scaling relationships and the statistical behaviour of ratio-based normalization. Across all age and sex groups, correlations between HW, BW, and TL were negligible to weak, indicating substantial deviations from proportionality. Simulations demonstrated that ratio-based normalization can generate misleading results, including spurious or reversed group differences, when proportionality assumptions are violated. Ratios were consistent with linear and allometric models only under strictly proportional conditions, characterized by regression lines passing through the origin. Linear models with covariate adjustment and allometric scaling provide more robust and biologically meaningful frameworks for organ weight analysis. Ratio-based normalization should be avoided unless key mathematical assumptions are met.

## Introduction

Heart weight (HW) is a fundamental parameter in both clinical cardiology and experimental mouse research, providing insights into structural and functional changes in the heart. In humans, increased HW, often indicative of hypertrophy, is a hallmark of pathological conditions such as hypertension and heart failure, associated with adverse clinical outcomes^1^. Accurate assessment of HW is essential for distinguishing pathological remodelling from normal physiological variants.

However, interpretation of HW is complicated by its dependency on body weight (BW). Substantial overlap between normal and abnormal HW ranges, driven in part by variation in body dimensions, has long challenged clinical and experimental comparisons^2,3^. These challenges have motivated the widespread use of normalization strategies intended to adjust HW for body size.

In mouse studies, HW is most commonly normalized to BW (HW/BW)^4^. Normalization to tibia length (HW/TL) has also gained popularity, as TL reflects skeletal growth and is less influenced by short-term changes in body mass^5^. Both approaches are routinely used to infer whether observed differences in HW reflect intrinsic cardiac changes or differences in overall body size^6^.

Despite their ubiquity, ratio-based normalization methods rely on a strong and often untested mathematical assumption: that the relationship between HW and the scaling variable is strictly linear and proportional^7–10^. When this assumption is violated, ratios can introduce bias, a phenomenon known as pseudo-indexing^11^. These concerns highlight the need for a systematic evaluation of normalization practices under biologically realistic conditions.

The aim of this study was to assess whether commonly used ratio-based normalization approaches are statistically and biologically appropriate for comparing HW across groups. Using a large, standardized dataset with more than 25,000 C57BL/6N wildtype control mice generated by the International Mouse Phenotyping Consortium, we sought to characterize empirical scaling relationships between HW, BW and TL across sex and age and to evaluate the implications of violating proportionality assumptions.

Our overarching hypothesis was that HW does not scale proportionally with BW in healthy mice and that the use of ratio-based normalization may therefore lead to misleading conclusions in group comparisons.

## Methods

### The international mouse phenotyping consortium

The International Mouse Phenotyping Consortium (IMPC) is a global collaborative initiative involving 24 research institutions, aiming to generate and systematically phenotype knockout mouse lines for all protein-coding genes with human orthologs. Phenotyping is performed according to standardized protocols defined in the IMPReSS resource^12–16^.

### IMPC centres and data sources

Analyses were performed using IMPC data release 21.0. Heart weight (HW) and body weight (BW) data were provided by eight IMPC centres, while tibia length (TL) data were available from three centres (HMGU, ICS, KMPC) for early- and late-adult pipelines, and from BCM for the early-adult pipeline only. Ethical approvals for all contributing centres are listed below.

1. Baylor College of Medicine (BCM) (Institutional Animal Care and Use Committee approved license AN-5896).
2. German Mouse Clinic, Helmholtz Zentrum München (HMGU) (#144-10, 15-168)
3. The Jackson Laboratory (JAX) Institutional Animal Care and Use Committee approved licenses 14004, 11005, and 99066. JAX AAALAC accreditation number was 000096, NIH Office of Laboratory Animal Welfare assurance number was D16-00170).
4. Institute Clinique de la Souris, Mouse Clinical Institute (ICS) (#4789-2016040511578546v2).
5. RIKEN BioResource Research Center (RBRC) (Animal Care Committee approved animal use protocols 0153, 0275, 0277, and 0279).
6. University of California – Davis (UCD) (Institutional Animal Care and Use Committee approved animal care and use protocol number 19075. UCD AAALAC accreditation number is 000029, and the NIH Office of Laboratory Animal Welfare assurance number is D16-00272 # (A3433-01).
7. Seoul National University, Korea Mouse Phenotyping Center (KMPC) (KRIBB-AEC-19189).
8. The Centre for Phenogenomics, Toronto (TCP) (0275 and 0279).

### Animals

Data were obtained from male and female wild-type control mice on a C57BL/6N background (sub-strains as specified per centre): C57BL/6NCrl (HMGU, ICS and TCP); C57BL/6NJ (BCM, JAX), C57BL/6NJcl (RBRC) and C57BL/6NTac (HMGU, ICS, KMPC and RBRC). Mice were phenotyped either in the early-adult (EA; median 16 weeks, range 15–20 weeks) or late-adult (LA; median 60 weeks, range 55–81 weeks) pipeline. Animal welfare was routinely assessed throughout the phenotyping process.

### Heart and body weight and tibia length measurements

Heart weight, body weight, and tibia length were measured according to IMPC standard operating procedures as defined in the IMPReSS resource^16^.

In brief, body weight (in g) was recorded and then the mouse humanely euthanized. The mouse was placed in the supine position and exposed fur was wiped with 70% ethanol to control dander. Centre-specific complete necropsy and tissue collection according to technical SOPs were performed. This included removal of the heart by dissecting the aortic root immediately above the aortic valves and the superior vena cava above the atria. Adjacent mediastinal fat pads were carefully removed from the excised heart with forceps and emptying the heart of blood by repeatedly tapping the heart on a Kimwipe (absorbent pad) or surgical compress until the heart was totally empty of blood.

Heart weight (in mg) was recorded in the centre-specific database prior to immersing the heart in a fixative solution. All data were collected at a local workstation in the necropsy room (attached to a digital balance) and uploaded to the centre-specific pathology data capture system. Data were then uploaded to the IMPC data coordination centre for quality control, processing and analysis prior being released on the IMPC portal.

The tibia lengths (TL) were measured using a calliper after organ extraction. The mouse’s hind limb was bent, and the length measured from the midpoint of the kneecap to the ankle joint^17^. Tibia length measurements, in millimetres (mm), were obtained using electronic callipers, with values transferred automatically to the local data capture system either via direct electronic connection or wireless transmission, depending on the contributing centre. Data were then uploaded to the IMPC data coordination centre for quality control, processing and analysis prior being released on the IMPC portal.

### Statistical methods

Statistical analyses were performed specifically for the aims of this study to assess HR, BW and TL normalization models independently of the summary statistics and analytical methods used for general data presentation on the IMPC portal.

Data analysis was conducted using *R* (version 4.2.2, R Core Team 2022) with figures and tables produced using *ggplot2* and *ggpubr*. Statistical analyses were performed separately for two data types: *real data,* consisting of measurements collected from IMPC mice, and *simulated data,* consisting of randomly generated values drawn from a normal distribution in the modelling approach.

### Statistical analysis of empirical data - Real data

Visual methods, as well as formal statistical tests, specifically the Shapiro Wilk test^18^, were applied to test whether the scores of the individual parameters were normally distributed (results not shown). Given the large sample size, visual inspection was emphasized for interpretation. Data were stratified by age and sex and histograms for each parameter were plotted. Reference ranges were calculated based on median, 2.5th percentile, and 97.5th percentile. In addition, the mean, standard deviation, and sample size for each parameter were provided to reflect the data distribution. To explore the linear associations between HW, BW and TL, the Pearson correlation coefficient (Pearson’s *r*^19^) was calculated, stratified by sex and age. Relationship strength was defined according to user guidelines^20^ with thresholds of *0.00-0.10* (negligible), *0.10-0.39* (weak), *0.40-0.69* (moderate), *0.70-0.89* (strong) and *0.90-1.00* (very strong). The effects of sex (female vs male) and age (EA vs LA) were compared using identical statistical analyses. For each case, a two-tailed Student’s t-test (Student 1908) was performed, and the Cohen’s d effect size was calculated using the *effsize* package. Overinterpretation of statistical significance is common in large samples. Thus, we additionally calculated effect sizes to provide a more meaningful estimate of biological relevance.

### Simulation framework - Simulated data

In the simulation framework, *P*_*1*_ and *P*_*2*_ denote two generic quantitative biological parameters, such as an organ weight and a body size related measure. This abstraction was chosen to ensure that the modelling results are applicable beyond heart weight normalization. To compare two independent samples (Group 1 vs. Group 2) in the modelling, we used a t-test, assuming data drawn from a normal distribution. This test determines whether the population means of *P*_*1*_ and *P*_*2*_ differ between Group 1 and Group 2 (Table 1, Panel A and B). The functional forms used to generate *P*_*2*_ in the simulation cases were deliberately chosen to represent distinct and well-defined scaling scenarios rather than to model specific biological parameters. Case 1A represents a linear but non-proportional relationship, achieved by including a non-zero intercept, Case 1B represents a strictly proportional linear relationship with a zero intercept, and Case 1C represents statistical independence between *P*_*1*_ and *P*_*2*_. The specific numerical constants were selected for simplicity and to ensure values remained within a biologically plausible range. The conclusions of the simulation depend on the presence or absence of proportionality, not on the absolute parameter values.

**Table 1:** Simulated data from two independent samples (Group 1 and Group 2) were used in the modelling Cases 1A-1C to determine if their population distributions differ.

Simulation case	Group	Group size	Parameter 1	Parameter 2	Error term *ε*
Case 1A	Group 1	n=10	N(35,3)	30 + (1.5 * Parameter 1) + ε	N(0,3)
	Group 2	n=10	N(30,3)	30 + (1.5 * Parameter 1) + ε	N(0,3)
Case 1B	Group 1	n=10	N(35,3)	1.5 * Parameter 1 + ε	N(0,3)
	Group 2	n=10	N(30,3)	1.5 * Parameter 1 + ε	N(0,3)
Case 1C	Group 1	n=10	N(35,3)	N(80,5)	
	Group 2	n=10	N(30,3)	N(80,5)	

**Case**	**How *P***_***2***_** is determined**
1A	Related to *P*_*1*_, but not in a fixed proportion (sophisticated function)
1B	A simple multiple of *P*_*1*_ (proportional)
1C	Random, with no connection to *P*_*1*_ (independent)

Panel A: Detailed description of cases and Panel B: The dependence of Parameter 2 (*P*_*2*_) and 1 (*P*_*1*_).

The following simulation scenarios were tested:

- **Case 1A:** linear but *non-proportional* (non-zero intercept) relationship. Both *P*_*1*_ and *P*_*2*_ are significantly lower in Group 2 compared to Group 1.
- **Case 1B:** linear and *proportional* (zero intercept) relationship. Both *P*_*1*_ and *P*_*2*_ are significantly lower in Group 2 compared to Group 1.
- **Case 1C:** no relationship (independent variables). *P*_*1*_ and *P*_*2*_ are independent and exhibit a non-proportional, random relationship. *P*_*1*_ is significantly lower in Group 2, while *P*_*2*_ is comparable between the groups.

Importantly, the simulation scenarios were designed to illustrate structural properties of ratio-based normalization, rather than to reflect outcomes from a single stochastic realization. In Case 1C, although *P*_*1*_ and *P*_*2*_ are generated independently, a group difference in the denominator (*P*_*1*_) induces a systematic group difference in the ratio (*P*_*1*_/*P*_*2*_). This effect is inherent to ratio-based analyses under non-proportional relationships and is consistently observed across repeated simulations with identical assumptions.

### Linear model

A linear model is a statistical approach used to describe the relationship between a dependent variable (*Y*) and one or more independent variables (*X*)^21^, assuming this relationship can be expressed as a linear equation. In its simplest form, the model can be written as

$$Y=\alpha + \beta X+ \epsilon$$

where $\alpha$ is the intercept representing the expected value of *Y* when *X=0*, $\beta$ is the regression coefficient describing the change in *Y* per unit change in *X*, and $\epsilon$ denotes the error term.

Here a linear model was fitted using the *lm()* function in *R* to perform a simple linear regression between HW and BW.

### Allometric scaling

Allometric scaling follows a power-law relationship rather than a simple linear trend^22^. The allometric model is typically expressed as

$$Y=a{X}^{b}$$

Where *a* is the scaling constant and *b* is the scaling exponent.

After log-log transformation, the relationship can be written as

$$\mathrm{log}Y=\mathrm{log}a+b\mathrm{log}X$$

where the scaling exponent *b* is estimated as the slope of a linear regression on $\mathrm{log}Y$ on $\mathrm{log}X$.

Although the allometric model does not include an additive intercept in raw data space, this reflects a biologically motivated boundary condition: organ size approaches zero as body size approaches zero. After log–log transformation, the model includes an intercept term ($\mathrm{log}a$), which represents the scaling constant rather than an additive offset. Crucially, both the scaling and the exponent are estimated from the data. Proportionality is therefore evaluated through the estimated scaling exponent *b*, rather than imposed a priori, distinguishing allometric scaling from linear models without an intercept. In this study, the coefficient *b* indicates how HW scales with BW or TL. Allometric scaling relationships between HW and BW, as well as between HW and TL, were generated.

All models and *R*-code are openly available in our GitHub repository: https://github.com/ExperimentalGenetics/AllometricScaling.

## Results

Heart weight (HW) data collected by the IMPC contributing centres (data release DR 21.0) are available from 25,354 wildtype mice. In the EA population, 27 mice were excluded from analysis due to age inconsistency. The remaining 25,327 mice were stratified as presented in Table 2 and summarized below. All mice are from a C57BL/6N inbred substrain. Most mice (86.3% or 21,846) were tested at a median age of 16 weeks (designated as “early adult” or EA), while the remaining 13.7% (3,481) of mice were tested at a median age of 60 weeks (designated as “late adult” or LA). Sex is evenly distributed at both the EA and LA time points. Body weight (BW) data are available for all mice whereas tibia length (TL), a non-mandatory IMPC parameter, is available for 7,775 (35.6% of 21,846) EA and 1,431 (41.1% of 3,481) LA mice. Raw data was downloaded from the following link: https://www.mousephenotype.org/data/release. The total number of parameters reported varies slightly between mice and can be accessed in each table.

**Table 2:** Heart weight data were available from a total of 25,327 mice, stratified by sex and age at testing (EA = median of 16 weeks of age; LA = median of 60 weeks of age).

Age	EA, Early adult		LA, Late adult		
Sex	Females	Males	Females	Males	Total
Heart weight [mg]	11016	10830	1782	1699	25327
Tibia length [mm]	3890	3885	724	707	9206

Body weight was recorded for all mice; however, tibia length measurements were not uniformly available across the dataset.

### Evaluating data distribution: metrics and significance testing

**Fig. 1:**
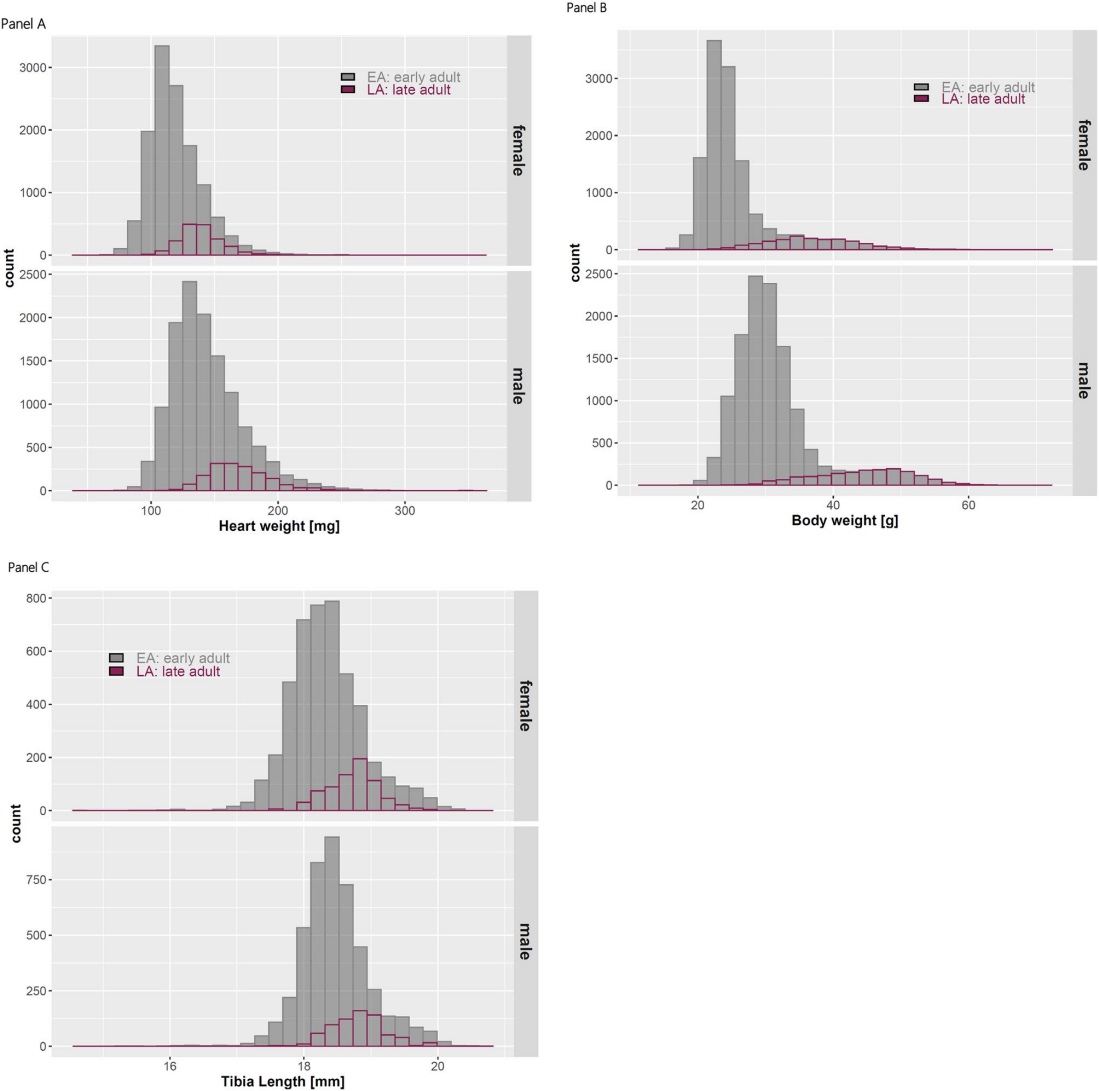
Histograms showing the distribution of heart weight [mg], body weight [g] and tibia length [mm] data for EA (grey) and LA (purple) mice, stratified by sex. These calculations are based on data from eight contributing IMPC centres. Panel A depicts heart weight, Panel B body weight and Panel C tibia lengths for EA (grey) and LA (red) mice, stratified by sex.

The distribution of data was assessed using histograms for HW, BW and TL stratified by sex and age (Figure 1, Panels A-C). Calculated ranges using mean ± standard deviation (SD), as well as median and 95% reference ranges (2.5th and 97.5th percentiles) were additionally provided for consistency (Table 3). Histograms indicated that all parameters were approximately normally distributed in both EA and LA groups, for male and female mice, while also highlighting minor deviations from normality.

**Table 3:** Sex-specific heart weight [mg], body weight [g] and tibia length [mm] data for EA (early adult) and LA (late adult) mice calculated as ranges of mean ± SD and median with 95% reference ranges.

Age	Sex	Parameter [unit]	Mean	±SD	Median	2.5th Percentile	97.5th Percentile	Group size
EA	Female	Heart weight [mg]	115.9	19.2	113	86.5	164.8	11027
		Body weight [g]	23.7	2.6	23.4	19.3	30	11059
		Tibia length [mm]	18.3	0.6	18.2	17.4	19.7	3894
	Male	Heart weight [mg]	139.5	26.4	134.9	101.2	207	10846
		Body weight [g]	29.4	3.5	29.4	22.9	36.6	10871
		Tibia length [mm]	18.4	0.5	18.4	17.5	19.7	3891
LA	Female	Heart weight [mg]	139.5	17.7	137.7	110.4	179.5	1782
		Body weight [g]	37.2	6.9	36.6	25.3	52.2	1789
		Tibia length [mm]	18.7	0.4	18.8	18	19.4	724
	Male	Heart weight [mg]	169.8	26	166.8	129.6	228.8	1699
		Body weight [g]	44.4	7.1	45.1	30.5	56.9	1708
		Tibia length [mm]	18.8	0.4	18.8	18.1	19.6	707

These calculations are based on data from eight contributing IMPC centres.

Figure 1 shows that EA males and females had similar distributions, with males having higher HW and BW and comparable TL. The same pattern was observed in the LA group. When comparing LA to EA by sex, both LA females and LA males showed higher HW, BW and TL values than their EA counterparts (Figure 1 and Table 3).

For each age group (EA and LA), sex-specific differences were evaluated using independent two-tailed *t*-tests, and Cohen’s *d* was calculated to quantify effect sizes. In both the EA and LA populations, HW, BW, and TL differed significantly (all *p≤0.001*), with group-level summary statistics and effect sizes reported in Table 4. Moreover, for all parameters, the corresponding Cohen´s d value revealed large effect sizes between sexes for HW (*1.03-1.37*) and BW (*1.02-1.87*), whereas TL showed minor (*0.29*) to negligible (*0.17*) effect sizes in EA and LA mice, respectively (Table 4, Panel A).

**Table 4:** Sex-specific differences in heart weight (HW), body weight (BW), and tibia length (TL) were evaluated in early adult (EA) and late adult (LA) groups.

Parameter			
Females vs Males	Age	Cohens´d	p-value
HW	EA, median 16 weeks	-1.03	p<0.001
	LA, median 60 weeks	-1.37	p<0.001
BW	EA, median 16 weeks	-1.87	p<0.001
	LA, median 60 weeks	-1.02	p<0.001
TL	EA, median 16 weeks	-0.29	p<0.001
	LA, median 60 weeks	-0.17	p=0.001

Early adult vs Late adult	Sex		
HW	Females	-0.24	p<0.001
	Males	-1.15	p<0.001
BW	Females	-3.82	p<0.001
	Males	-3.57	p<0.001
TL	Females	-0.90	p<0.001
	Males	-0.77	p<0.001

Two-tailed t-tests showed significant differences (*≤.001*) for all parameters, with large effect sizes for HW and BW and minor to negligible effects for TL based on Cohen’s d values (Panel A). Age-related comparisons also revealed significant differences (*≤.001*) with medium to large effect sizes for HW, BW, and TL, stratified by sex (Panel B).

Two different age groups, i.e. median age of 16-weeks (minimum 15 and maximum 20 weeks) old EA mice and median age of 60 weeks (minimum 55 and maximum 81 weeks) old LA mice, made it possible to explore the effect of age on HW, BW and TL, stratified by sex. *P*-values *≤.001* were reached for all parameters, indicating high statistical significance. The corresponding Cohen’s d effect size values revealed medium to large, standardized effect sizes in HW, BW and TL (Table 4, Panel B).

### Examining correlations: heart weight in relation to body weight and tibia length

Pearson correlation coefficients (*r*) were calculated to assess linear associations between HW and either BW or TL, stratified by sex and age. Female data are placed directly next to male data for ease of visualization.

**Fig. 2:**
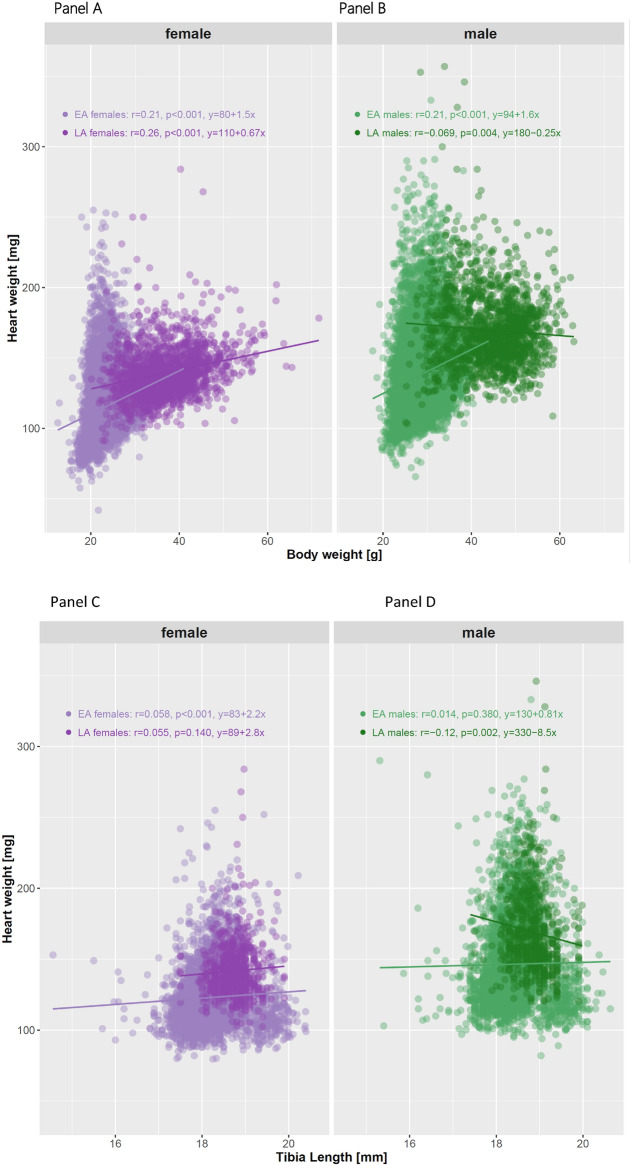
The linear models in the plot are represented by the regression equations displayed on each Panel. These equations describe the relationship between the independent variable (body weight [g] or tibia length [mm]) and the dependent variable (heart weight [mg]) for both female and male groups. The data are stratified by sex (female vs. male) and age group (early adult [EA] vs. late adult [LA]). Panel A: HW with BW in EA (*r=0.21*) and LA (*r=0.26*) female populations; Panel B: HW with BW in EA (*r=0.21*) and LA (*r=-0.069*) male populations. Panel C: HW with TL in EA (*r=0.058*) and LA (*r=0.055*) for females; Panel D: HW with TL in EA (*r=0.014*) and LA (*r=-0.12*) for males.

Figure 2 shows distinct distribution clusters with regression lines for EA and LA groups stratified by sex. Negligible to weak standardized Pearson correlations were obtained for both comparisons in the EA population with HW (mg) and BW (g) showing *r=0.21* for females and *r=0.21* for males (Figure 2, Panels A & B). In the LA population, the correlations were *r=0.26* for females and *r=-0.069* for males (Figure 2, Panels A & B), indicating a weak or negligible correlation between HW and BW; the HW (mg) to TL (mm) ratio reached in the EA population *r=0.058* for females and *r=0.014* for males (Figure 2, Panels C & D), whereas in the LA population it was *r=0.055* for females and *r=-0.12* for males (Figure 2, Panels C & D). Comparing EA and LA populations, the relationship flattens over time, indicating a non-linear trend across the lifespan from EA (median 16 weeks) to LA (median 60 weeks) in healthy C57BL/6N substrain mice (Figure 2, Panels A-D).

### Unveiling biases: modelling the limitations of ratio-based analyses in non-proportional relationships

Following on from the non-proportional relationship shown here, we aim to demonstrate through simulation-based modelling that using ratios to compare groups can lead to mathematically invalid or misleading inferences when proportionality assumptions are violated. We conducted a simulation study using artificial data to assess the performance of ratio-based normalization for detecting group differences under different assumptions about the functional relationships between variables. Simulated datasets were generated for two groups, Group 1 and Group 2, each consisting of 10 samples (Table 1, Panel A). The values for two parameters, Parameter 1 and Parameter 2, were used to reflect modelling scenarios across different cases (Table 1, Panel B). Parameter 1 (*P*_*1*_) was drawn from a normal distribution. Depending on the case, Parameter 2 (*P*_*2*_) was either derived from *P*_*1*_ through a defined relationship (non-linear or proportional) or independently sampled from a normal distribution with predefined mean and standard deviation.

- Case 1A: *P*_*2*_ is related to *P*_*1*_ but in a non-proportional way. This suggests a linear relationship such as: *P*_*2*_ = *f (P*_*1*_*)* + $\epsilon$  where *f (P*_*1*_*)* is a non-proportional function (*P*_*2*_≠c⋅*P*_*1*_), and $\epsilon$ is an error term.
- Case 1B: *P*_*1*_ is approximately proportional to *P*_*2*_ with *c* the constant scaling factor, expressed as a linear relationship *P*_*2*_ = *c*⋅*P*_*1*_+ $\epsilon$, where $\epsilon$  is an error term.
- Case 1C: *P*_*1*_ and *P*_*2*_ are randomly drawn from normal distributions, independent from each other: *P*_*1*_ = *N(μ*_*1*_*, σ*_*1*_^*2*^*), P*_*2*_ = *N(μ*_*2*_*, σ*_*2*_^*2*^*)*.

A t-test was used to compare group means, with significance evaluated at a threshold of *≤.05*. Table 1 provides model details.

**Fig. 3:**
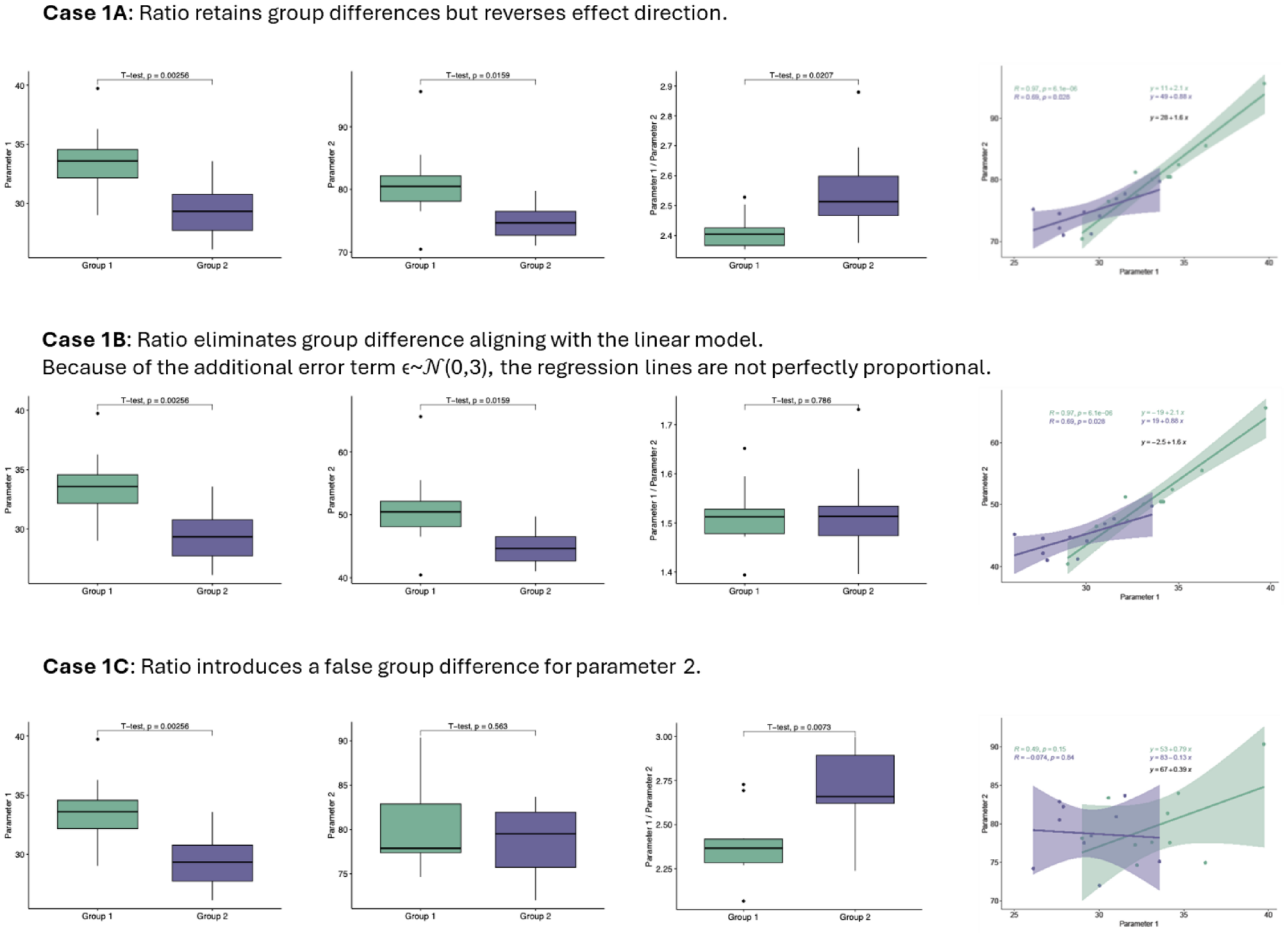
Modelling demonstrates the mathematical inconsistency of non-proportional relationships when comparing groups. Simulated datasets for two groups (n=10 per group; total N=20) were generated with values for two parameters randomly drawn from a normal distribution. Colour codes are as follows: green for Group 1 and purple for Group 2.

The results in Fig. 3 and Table 5 show that in Case 1A, applying the ratio of *P*_*1*_ to *P*_*2*_ retains a statistically significant difference between Group 1 and Group 2 (*≤.05*) but significantly reverses the direction of the effect compared to the raw data. This indicates that the ratio causes distortion under these conditions.

**Table 5:** Linear regression results from simulated two-group datasets (n = 10 per group; total N = 20) illustrating the limitations of ratio-based comparisons under non-proportional relationships.

Case	Coefficient	Estimate	CI Lower	CI Upper	P-Value	Total Size	R^2^	Adjusted R^2^
1A	Intercept	25.01	9.09	40.93	0.004	N=20	0.837	0.818
	Parameter 1	1.67	1.2	2.14	<0.001			
	Group	0.7	-2.37	3.77	0.637			
1B	Intercept	-4.99	-20.91	10.93	0.517	N=20	0.837	0.818
	Parameter 1	1.67	1.2	2.14	<0.001			
	Group	0.7	-2.37	3.77	0.637			
1C	Intercept	64.25	36.29	92.21	<0.001	N=20	0.094	-0.012
	Parameter 1	0.47	-0.36	1.29	0.25			
	Group	0.78	-4.62	6.18	0.763			

Coefficients, lower and upper 95% confidence intervals (CI), p-values, and model fit statistics (R^2^, adjusted R^2^) are reported. Valid ratio-based inference is observed only under proportional conditions in Case 1B.

In contrast, in Case 1B, *P*_*1*_ and *P*_*2*_ exhibit a linear and strictly proportional relationship. As expected from a purely mathematical perspective, *P*_*2*_ scales proportionally with *P*_*1*_ and therefore dominates the denominator of the ratio. This case serves as a control scenario, illustrating that ratio-based normalization does not introduce a mathematical inconsistency when proportionality holds. However, the ratio targets a different estimand than the absolute group difference in *P*_*1*_. Consequently, the apparent elimination of the group difference reflects a change in the scale of inference rather than an error or reversal. This example highlights that ratio-based normalization is only appropriate when proportionality holds and when the estimand of interest is explicitly defined.

In Case 1C, *P*_*1*_ and *P*_*2*_ are simulated independently and therefore exhibit a non-proportional relationship, with no true group difference in *P*_*2*_. Nevertheless, calculating the ratio *P*_*1*_*/P*_*2*_ yields a statistically significant group difference. This effect does not arise from random variation in a single simulation instance but reflects a systematic property of ratio-based analyses when the denominator differs between groups. Because the regression relationship between *P*_*1*_ and *P*_*2*_ does not pass through the origin, a fundamental assumption required for valid ratio normalization is violated. Under these conditions, the ratio inherits group-level information from the denominator, leading to spurious inference despite the absence of a true effect in the numerator. This finding further demonstrates that ratio-based normalization is invalid when proportionality assumptions are not satisfied.

Applying this simulated modelling approach to our data set, the conclusions remain identical. This implies that regardless of the body size parameter considered - whether TL or BW - the fundamental methodological challenges and scaling issues persist.

Simple ratio-based normalization methods often do not capture the underlying biological relationships. In comparison, linear and allometric models offer a more robust and interpretable analytical framework for group comparisons.

### Beyond ratios: a linear modelling approach for more robust biological scaling analysis

A linear model (LM) provides a simplified but powerful way to analyse and predict relationships between variables in both statistics and scientific research. Here, an LM assumes a direct, additive relationship between HW and BW, allowing for the estimation of changes in HW as a function of BW. This approach accounts for variability by including an intercept and a slope term, making it a more flexible alternative to simple ratio-based normalization. Fig. 2 shows the LM with regression lines for the relationship between HW and BW in early adult (EA) and late adult (LA) females (Panel A), and in EA and LA males (Panel B). Analogously, Panels C and D show the relationship between HW and TL for EA and LA females and males. Sex-specific relationships between heart weight, body weight, and tibia length are shown for each group. Furthermore, differences in estimated slope ($\beta$) suggest potential sex-specific differences in the relationship between parameters. Most equations (Panels A-C) have positive slopes, meaning HW increases as BW or TL increases. However, in Panel D, the male TL has a negative slope ($\beta$*=-8.5*), indicating a weak inverse association in the LA male population, which may reflect increased variability, sampling effects, or deviations from linearity in this subgroup.

In summary, the LM is visualized in Fig. 2 through regression equations and their corresponding trend lines, highlighting how HW depends on BW or TL in both males and females. The variation in slopes suggests sex-specific linear relationships.

### From linear models to allometric scaling: a scientific transition

The purpose of applying linear and allometric models is not to identify a universally superior functional form, but to explicitly model scaling relationships without imposing the proportionality assumptions inherent in ratio-based normalization. We initially modelled the relationship between HW and BW using a linear regression model, assuming a constant rate of change. However, organ weights often do not scale linearly with body weight^24–26^.

To account for nonlinear growth patterns, we adopted an allometric scaling model, which is commonly used in biological systems to describe how physiological traits change with body size^27^.

The allometric exponent *b* provides insights into the growth rate of HW relative to BW. When *b=1*, isometric scaling occurs, meaning that HW increases proportionally to BW. A value of *b<1* suggests negative allometry, where HW increases at a slower rate than BW, while *b>1* indicates positive allometry, where HW increases at a faster rate than BW.

**Fig. 4:**
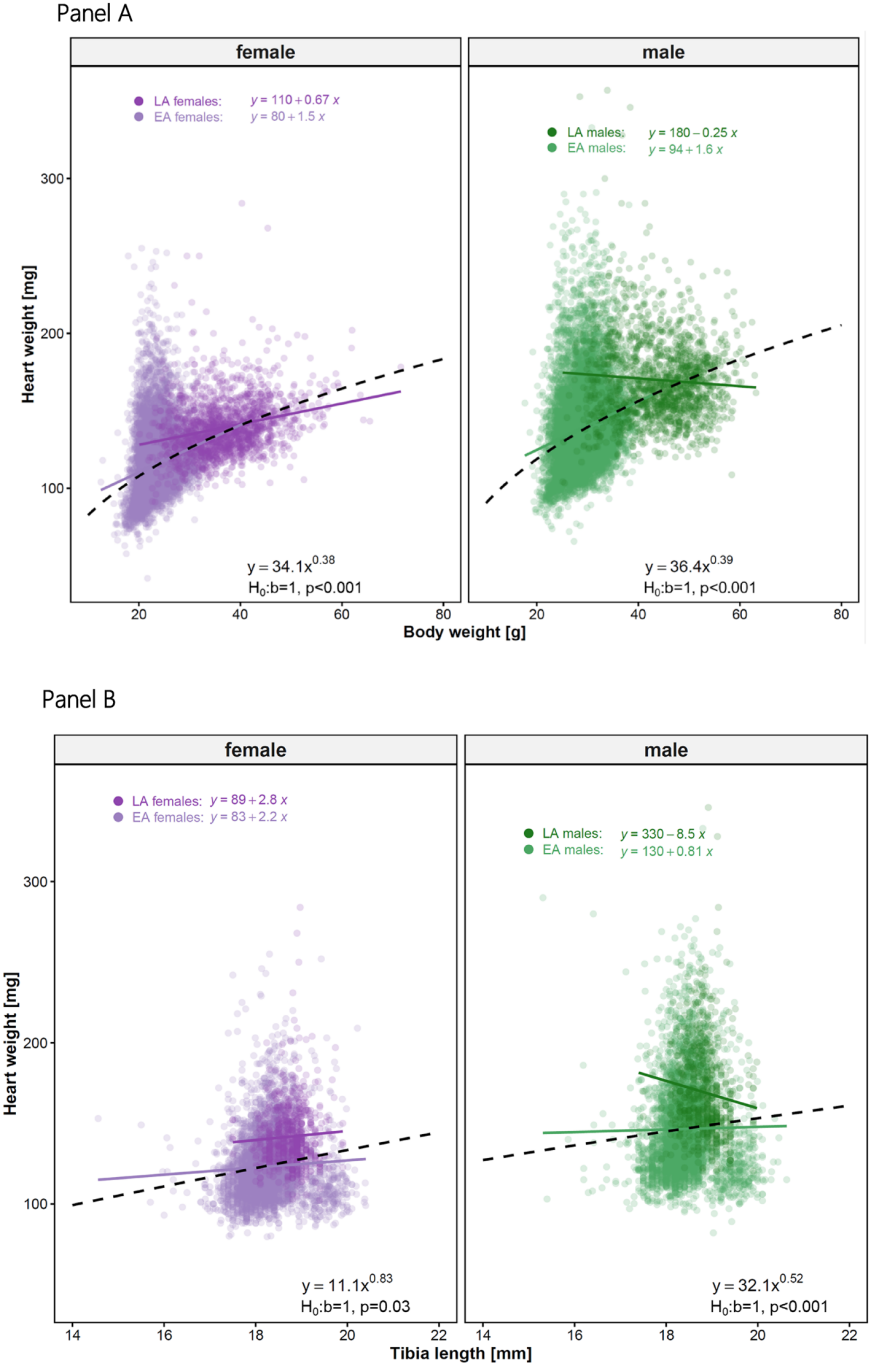
Allometric model for body weight [g] and heart weight [mg] represented by the regression equations. Data are stratified by sex and age. The allometric scaling exponent (*b*) and corresponding *p*-values testing deviation from isometric scaling (*b=1*) are indicated in each panel. Panel A: HW with BW in EA and LA populations split by sex. Panel B: HW with TL in EA and LA populations split by sex.

Figure 4 shows that our findings (*b=0.38* for females, *b=0.39* for males, Panel A) indicate negative allometry, meaning HW increases at a slower rate than BW or TL (*b=0.83* for females, *b=0.52* for males*, Panel B)* in a healthy wildtype population. This shift from a linear to an allometric approach provides a more biologically meaningful representation of cardiovascular scaling.

### Scientific justification for using allometry

The observed allometric exponents in our study for BW, *b=0.384±0.007* for females and *b=0.395±0.008* for males, indicate negative allometry (*b<1*) and differ significantly from isometric scaling (H_0_: *b=1*; *t(12521)=-74.75*, *p<0.001* for males; *t(12792)=-92.79*, *p<0.001* for females); for TL *b=0.831±0.078* for females and *b=0.523±0.100* for males, indicate negative allometry (*b<1*) and differ significantly from isometric scaling (H_0_: *b=1*; *t(4585)=-4.76*, *p<0.001* for males; *t(4605)=-2.17*, *p=0.03* for females), consistent with previous findings in cardiovascular physiology^27^. For example, studies such as Prothero^28^ and Calder^29^ have shown that mammalian heart weight typically scales with body weight with exponents below 1, reflecting negative allometry as animals increase in size. This suggests that, according to allometric scaling laws, as body size increases, the heart does not grow at the same rate but rather adapts to maintain efficiency in the circulatory function. Such relationships align with broader biological scaling principles, such as those described by Kleiber and Cole^30^, which describe how metabolic rates scale sublinearly with body mass.

## Discussion

The results presented here, supported by the extensive dataset provided by the International Mouse Phenotyping Consortium, emphasize the power of large-scale, standardized data to address methodological questions in quantitative biology. Our study highlights the limitations of ratio-based analyses in allometric datasets and emphasizes the importance of using linear models with covariate adjustment or, where appropriate, allometric scaling models to generate robust and interpretable results.

### Biological context: allometry and ratios

In normal physiological conditions, organ weights, such as heart weight (HW), do not always exhibit strong linear and proportional relationships with metrics like body weight (BW) or tibia length (TL). This study shows that in wildtype C57BL/6N mice, correlations between HW and BW, and HW and TL, are weak to negligible across sex and age groups. Such weak non-proportional relationships inherently undermine the validity of ratio-based normalization (e.g., HW/BW or HW/TL) for detecting group differences, as these ratios assume proportional scaling between variables - an assumption not supported by the data.

Importantly, the observed negative allometry is consistent with previous reports describing sublinear scaling of cardiac and other organ masses relative to body size across species and physiological contexts^31^.

The observed variability in HW relative to BW and TL also reflects the biological reality that organ development and body size are influenced by different environmental and physiological factors. This underscores the need for analytical approaches that account for these complexities without introducing artifacts^32^.

### Modelling study: pitfalls of ratio-based normalization

Through simulated data, we demonstrated how using ratios under weak linear or non-proportional relationships can lead to misleading group-level inferences when comparing groups. In Case 1A, applying a ratio preserved statistically significant group differences but reversed the direction of the effect, leading to misleading conclusions. Conversely, in Case 1B, where specific assumptions (e.g., a regression line passing through origin) were met, the ratio produced accurate results comparable to those from linear models. However, Case 1C demonstrated that ratios can artificially create significant differences between groups even when none exist, a direct consequence of invalid proportionality assumptions.

These findings reinforce that ratio-based comparisons between groups are only appropriate under specific conditions, namely when the relationship between variables is both linear and proportional, that is when the regression line passes through the origin. Otherwise, ratios can distort results, as demonstrated in this study.

### Rethinking ratios: considerations for and limitations of large-scale phenotyping

The IMPC dataset, encompassing over 25,000 wildtype mice, offers unmatched statistical power and granularity for systematically investigating allometric relationships. Utilizing this extensive resource, we demonstrate that linear or power-law based models provide more robust and interpretable inferences than ratio-based approaches in analysing allometric data. Unlike ratios, these models maintain the integrity of the underlying biological relationships, adjust for confounding variables, and provide statistically robust and biologically meaningful conclusions.

No study is without limitations. In our case, we cannot exclude that the large-scale multi-centre setup introduces centre effects and parameter variations that may influence the results. Although normalization to body surface area is widely used in clinical cardiology, this parameter is not routinely measured within the IMPC phenotyping pipeline and was therefore not available for analysis in the present study. Looking ahead, including mutant mouse lines and distinguishing whole-body weight from body composition will help assess non-allometric scaling relevant for compensatory or pathological hypertrophy in the heart. We modelled three scenarios here, but they do not cover all possible real-world situations, so results may vary depending on the biological context. In consequence, the key takeaway is that careful visual inspection and preliminary data exploration are essential for the selection of appropriate models to draw meaningful conclusions.

### Theoretical assumptions underlying ratio normalization

Ratio-based normalization is theoretically valid only when the response variable scales strictly proportionally with the normalization variable, such that the relationship is linear and passes through the origin. Under these conditions, the ratio estimates the proportionality constant and is equivalent to a regression model constrained to have no intercept.

In practice, however, these assumptions are rarely met. Non-zero intercepts, nonlinear scaling, heteroscedasticity, and measurement error in the scaling variable all compromise the validity of ratio-based comparisons. When the underlying relationship does not pass through the origin, ratio normalization can generate apparent group differences that reflect differences in the distribution of the scaling variable rather than true effects on the outcome. The statistical limitations of ratio standards and the advantages of covariate-based and allometric approaches have been extensively discussed in the literature^33–36^.

Measurement error in the scaling variable further exacerbates these biases by attenuating associations and distorting ratio estimates, consistent with well-established error-in-variables effects^37^. Although analytical corrections exist, they rely on additional assumptions and are difficult to apply in large, heterogeneous phenotyping datasets. In response to these limitations, alternative approaches to ratio-based normalization have been proposed, including dimensional indexing and the use of matching units to preserve physical interpretability across traits^11^.

For these reasons, the present study favors an empirical and simulation-based approach that explicitly evaluates the consequences of violating proportionality assumptions under biologically realistic conditions. This framework allows us to assess the robustness of different analytical strategies without relying on idealized model assumptions.

### Guiding future research: key recommendations based on findings

- Ratio-based comparisons between groups should be avoided unless the relationship between parameters is strictly linear and proportional, with a regression line passing through the origin.
- Including a scatterplot representation of the data should be considered a minimal prerequisite, as it offers a meaningful way to explore the data structure before applying statistical models.
- Linear and power-law models with covariate adjustments should be the preferred methods for analysing allometric relationships, as they control confounders and accommodate certain non-proportionality.
- The use of large-scale datasets, such as those generated by the IMPC, should be encouraged to validate analytical approaches and ensure reproducibility across diverse biological contexts.

## Conclusion: Why the shift matters

Transitioning from ratio-based normalization to model-based approaches provides a more biologically meaningful interpretation of how heart weight varies with body weight. Linear models with covariate adjustment and allometric scaling explicitly model scaling relationships and accommodate nonlinear growth dynamics, thereby capturing physiological constraints across different body sizes without imposing unsupported proportionality assumptions.

The key conclusion of this study is that ratio-based normalization should not be used for group comparisons unless strict proportionality between traits can be demonstrated. When proportionality is violated, ratios can yield misleading or spurious inferences, whereas linear and power-law models provide statistically valid and interpretable alternatives. Importantly, the choice between linear and allometric models should be guided by the structure of the data and biological considerations, rather than by visual goodness-of-fit alone.

Finally, we note that the scope of these conclusions is shaped by our computational choices: effect sizes and statistical inference may vary with model specification, distributional assumptions, and the handling of influential observations. Although our approach is well suited to the IMPC framework, alternative strategies (e.g., robust, hierarchical, or differently parameterized nonlinear models) could yield quantitatively different estimates; thus, our conclusions are most directly applicable to the modeling framework evaluated here and should be complemented by targeted sensitivity analyses.

By leveraging the IMPC’s extensive, standardized phenotyping data, this study highlights fundamental limitations of ratio-based analyses and provides practical guidance for improving analytical rigor in quantitative biology, ultimately advancing our understanding of complex biological systems.

## Data Availability

All data used in this study are publicly available from the International Mouse Phenotyping Consortium (IMPC) data releases^23^.

## References

[1] Martin, T. G., Juarros, M. A. & Leinwand, L. A. Regression of cardiac hypertrophy in health and disease: Mechanisms and therapeutic potential. *Nat. Rev. Cardiol.* **20**, 347–363. 10.1038/s41569-022-00806-6 (2023). 36596855 10.1038/s41569-022-00806-6 PMC10121965

[2] Dewey, F. E., Rosenthal, D., Murphy, D. J., Froelicher, V. F. & Ashley, E. A. Does size matter?. *Circulation* **117**, 2279–2287. 10.1161/CIRCULATIONAHA.107.736785 (2008). 18443249 10.1161/CIRCULATIONAHA.107.736785

[3] Pfaffenberger, S. et al. Size matters! impact of age, sex, height, and weight on the normal heart size. *Circ. Cardiovasc. Imaging.* **6**, 1073–1079. 10.1161/CIRCIMAGING.113.000690 (2013). 24014823 10.1161/CIRCIMAGING.113.000690

[4] Doevendans, P. A., Daemen, J. M. ., de Muinck, E. D. & Smits, J. F. Cardiovascular phenotyping in mice. *Cardiovasc. Res.* **39**, 34–49. 10.1016/s0008-6363(98)00073-x (1998). 9764188 10.1016/s0008-6363(98)00073-x

[5] Lindsey, M. L., Kassiri, Z., Virag, J. A. I., de Castro Brás, L. E. & Scherrer-Crosbie, M. Guidelines for measuring cardiac physiology in mice. *Am. J. Physiol. Heart Circ. Physiol.* **314**, H733-h752. 10.1152/ajpheart.00339.2017 (2018). 29351456 10.1152/ajpheart.00339.2017 PMC5966769

[6] Lerchenmüller, C. et al. Restoration of cardiomyogenesis in aged mouse hearts by voluntary exercise. *Circulation* **146**, 412–426. 10.1161/CIRCULATIONAHA.121.057276 (2022). 35862076 10.1161/CIRCULATIONAHA.121.057276 PMC9357140

[7] Karp, N. A., Segonds-Pichon, A., Gerdin, A. K., Ramírez-Solis, R. & White, J. K. The fallacy of ratio correction to address confounding factors. *Lab. Anim.* **46**, 245–252. 10.1258/la.2012.012003 (2012). 22829707 10.1258/la.2012.012003 PMC4152922

[8] Simkus, P. et al. Limitations of cardiothoracic ratio derived from chest radiographs to predict real heart size: Comparison with magnetic resonance imaging. *Insight. Imaging.* **12**, 158–158. 10.1186/s13244-021-01097-0 (2021). 10.1186/s13244-021-01097-0 PMC8566609 34731329

[9] Oellrich, A. et al. Reporting phenotypes in mouse models when considering body size as a potential confounder. *J. Biomed. Semantics.* **7**, 2–2. 10.1186/s13326-016-0050-8 (2016). 26865945 10.1186/s13326-016-0050-8 PMC4748495

[10] Hoit, B. D. & Litwin, S. E. The new normal: How should we assess cardiac chamber sizes and proportionality across the full spectrum of body sizes with varying degrees of adiposity?. *J. Am. Soc. Echocardiogr.* **35**, 151–153. 10.1016/j.echo.2021.11.013 (2022). 34875314 10.1016/j.echo.2021.11.013

[11] Hagdorn, Q. A. J. et al. A novel method optimizing the normalization of cardiac parameters in small animal models: The importance of dimensional indexing. *Am. J. Physiol. Heart Circ. Physiol.* **316**, H1552–H1557. 10.1152/ajpheart.00182.2019 (2019). 30978120 10.1152/ajpheart.00182.2019

[12] Dickinson, M. E. et al. High-throughput discovery of novel developmental phenotypes. *Nature* **537**, 508–514. 10.1038/nature19356 (2016). 27626380 10.1038/nature19356 PMC5295821

[13] Groza, T. et al. The international mouse phenotyping consortium: comprehensive knockout phenotyping underpinning the study of human disease. *Nucl. Acid. Res.* **51**, D1038–D1045. 10.1093/nar/gkac972 (2022). 10.1093/nar/gkac972 PMC9825559 36305825

[14] Muñoz-Fuentes, V. et al. The international mouse phenotyping consortium (IMPC): A functional catalogue of the mammalian genome that informs conservation. *Conserv. Genet.* **19**, 995–1005. 10.1007/s10592-018-1072-9 (2018). 30100824 10.1007/s10592-018-1072-9 PMC6061128

[15] Brown, S. D. M., Chambon, P., de Angelis, M. H. & Eumorphia, C. EMPReSS: Standardized phenotype screens for functional annotation of the mouse genome. *Nat. Genet.* **37**, 1155. 10.1038/ng1105-1155 (2005). 16254554 10.1038/ng1105-1155

[16] IMPReSS: International Mouse Phenotyping Resource of Standardised Screens. https://www.mousephenotype.org/impress/ (2011).

[17] Silva, M. J., Brodt, M. D. & Hucker, W. J. Finite element analysis of the mouse tibia: Estimating endocortical strain during three-point bending in SAMP6 osteoporotic mice. *Anat. Rec. A Discov. Mol. Cell Evol. Biol.* **283**, 380–390. 10.1002/ar.a.20171 (2005). 15747345 10.1002/ar.a.20171

[18] Shapiro, S. S. & Wilk, M. B. An analysis of variance test for normality (complete samples). *Biometrika* **52**, 591–611. 10.2307/2333709 (1965).

[19] Pearson, K. Note on regression and inheritance in the case of two parents. *Proc. R. Soc. Lond.* **58**, 240–242 (1895).

[20] Schober, P., Boer, C. & Schwarte, L. A. Correlation Coefficients: Appropriate Use and Interpretation. *Anesth. Analg*. **126**, 1763–1768. 10.1213/ane.0000000000002864 (2018). 29481436 10.1213/ANE.0000000000002864

[21] Gelman, A. & Hill, J. Data analysis using regression and multilevel/hierarchical models 10.1017/CBO9780511790942 (Cambridge University Press, 2006).

[22] Schmidt-Nielsen, K. Scaling in biology: The consequences of size. *J. Exp. Zool.* **194**, 287–307. 10.1002/jez.1401940120 (1975). 811757 10.1002/jez.1401940120

[23] (IMPC)., I.M.P.C. IMPC data release https://www.mousephenotype.org/data/release (2011).

[24] Müller, M. J. et al. Effect of constitution on mass of individual organs and their association with metabolic rate in humans--A detailed view on allometric scaling. *PLoS. One.* **6**, e22732. 10.1371/journal.pone.0022732 (2011). 21818376 10.1371/journal.pone.0022732 PMC3144246

[25] Antoł, A. & Kozłowski, J. Scaling of organ masses in mammals and birds: Phylogenetic signal and implications for metabolic rate scaling. *ZooKeys* **982**, 149–159. 10.3897/zookeys.982.55639 (2020). 33239956 10.3897/zookeys.982.55639 PMC7652810

[26] Vea, I. M. & Shingleton, A. W. Network-regulated organ allometry: The developmental regulation of morphological scaling. *WIREs Dev. Biol.* **10**, e391. 10.1002/wdev.391 (2021). 10.1002/wdev.391 32567243

[27] Dawson, T. H. Allometric relations and scaling laws for the cardiovascular system of mammals. *Systems* **2**, 168–185 (2014).

[28] Prothero, J. Heart weight as a function of body weight in mammals. *Growth* **43**, 139–150 (1979). 510954

[29] Temerin, L. A. Size, function, and life history. By W.A. Calder III. Cambridge: Harvard University Press. 1984. xii + 431 pp., figures, tables, appendices, index. $32.50 (cloth). *Am. J. Phys. Anthropol.* **66**, 340–342. 10.1002/ajpa.1330660312 (1985).

[30] Kleiber, M. & Cole, H. H. Body size, growth rate and metabolic rate in two inbred strains of rats. *Am. J. Physiol.* **161**, 294–299. 10.1152/ajplegacy.1950.161.2.294 (1950). 15425648 10.1152/ajplegacy.1950.161.2.294

[31] Glazier, D. S. Beyond the ‘3/4-power law’: variation in the intra-and interspecific scaling of metabolic rate in animals. *Biol. Rev.* **80**, 611–662. 10.1017/S1464793105006834 (2005). 16221332 10.1017/S1464793105006834

[32] Vollmer, J., Casares, F. & Iber, D. Growth and size control during development. *Open. Biol.* **7**. 10.1098/rsob.170190 (2017). 29142108 10.1098/rsob.170190 PMC5717347

[33] Atchley, W. R., Gaskins, C. T. & Anderson, D. Statistical properties of ratios. I. empirical results. *Syst. Zool.* **25**, 137–148. 10.2307/2412740 (1976).

[34] Kronmal, R. A. Spurious correlation and the fallacy of the ratio standard revisited. *J. R. Stat. Soc. A Stat. Soc.* **156**, 379–392. 10.2307/2983064 (2018).

[35] Magnusson, W. E. Ratios, statistics, and physiological models: comment on packard and boardman. *Physiol. Zool.* **62**, 997–1000 (1989).

[36] Packard, G. C. & Boardman, Timothy J. The misuse of ratios, indices, and percentages in ecophysiological research. *Physiol. Zool.* **61**, 1–9. 10.1086/physzool.61.1.30163730 (1988).

[37] McArdle, B. The structural relationship: Regression in biology. *Can. J. Zool.* **66**, 2329–2339. 10.1139/z88-348 (2011).

